# Changes in biochemical compositions and salinity tolerance responses of different bread wheat varieties cultivated in an arid and semi-arid climate

**DOI:** 10.1038/s41598-025-28944-0

**Published:** 2025-12-29

**Authors:** Saeed Norouzi, Gholamali Akbari

**Affiliations:** Department of Agronomy and Plant Breeding Sciences, College of Aburaihan, University of Tehran, Tehran, Pakdasht, Iran

**Keywords:** Enzymatic/non-enzymatic activities, Gluten index, Grain protein, Grain yield, Salt tolerance, Superior cultivars, Physiology, Plant sciences

## Abstract

The present study aimed to investigate the differential responses of several wheat cultivars under saline conditions through two complementary experiments, a laboratory-based Petri dish test and a field trial. Therefore, the effects of salinity levels (control, 4, 8, and 12 dS·m^−1^) were firstly studied on seed germination indices and some growth-related parameters of six bread wheat cultivars/new promising lines (e.g., cultivars of Chamran-2, Mehrgan, Marvdasht, and Narin, as well as new promising lines of MS-89-13 and MS-90-13) using a factorial based on the completely randomized design in the Petri test for ten days in three replications. Subsequently, different responses of the superior cultivars/lines selected were evaluated under both normal and saline field conditions through a combined analysis using a randomized complete block design, conducted over the 2020–2021 and 2021–2022 growing seasons with three replications. The Petri data showed that salinity levels negatively influenced germination indices, with the highest germination percentage, optimal T50 values, longest shoot length, and highest leaf protein content observed under the control (non-saline) treatment across all cultivars. Among cultivars, the Chamran-2 cultivar achieved the highest germination percentage, shoot length, and leaf protein content, and the lowest T50 value. Additionally, the minimum values of root length and root length stress tolerance index traits were observed for the interaction of MS-89-13 promising line × 12dS·m^−1^ salinity level. Field experiment data revealed that the highest values for plant height, 1000-grain weight, grain and biological yields, pigment contents, grain protein, wet gluten, and gluten index were recorded in plants grown under normal conditions during the second year of the study. Chamran-2, and then Mehregan had more proper conditions and had longer plants, heavier grain weight, and higher grain and biological yields. However, the maximum values for wet gluten and gluten index were obtained for Mehregan and Narin cultivars, respectively. The highest straw yield was obtained under the Chamran-2 cultivar × Normal farm × Second year interaction. The highest catalase activity was recorded for saline conditions and the first year of the experiment, and the highest superoxide dismutase activity was observed for the Narin cultivar × Saline conditions × Second year interaction. Eventually, considering the predominant characteristics of the field experiments, Chamran-2 and Mehrgan cultivars can be cultivated in the southern regions of Iran and similar areas as a reference.

## Introduction

Plants are subjected to various biotic and abiotic stresses worldwide, including salinity, drought, light, heavy metals, temperature extremes, pathogens, or a combination of all these factors^1–4^. Among these, abiotic stresses, particularly salinity, significantly reduce crop productivity^5,6^. Meanwhile, salinity primarily resulting from high levels of sodium chloride and other soluble salts such Na^+^, Cl^–^, Ca^2+^, Mg^2+^, and others in soils or irrigation water sources, restricts plant growth, development, and productivity^7–11^. It has also been estimated that 20% of total cultivated lands and about 33% of irrigated agricultural lands worldwide are affected by salinity, with the problem increasing daily^12,13^, especially in arid and semi-arid regions due to salt accumulation^14,15^. It was also stated that salt stress, based on its intensity and duration, induced numerous changes in plant physiological and metabolic processes^16^ and led to secondary disorders, such as oxidative, osmotic, ionic imbalance, and other stressful conditions due to inducing ROS formation in plant cells and cellular components^17–19^. In this way, the secondary stresses induced by high salt levels and high tendency of Na + to replace K + cause disruptions in plant morphology, plant physiology, reducing water absorption from the roots, stomata closing to decrease water loss via transpiration, limiting CO_2_ absorption, and photosynthesis performance^20–26^. In this regard, numerous enzymatic and non-enzymatic antioxidants, including superoxide dismutase (SOD), ascorbate peroxidase, glutathione reductase, dehydro-ascorbate reductase, catalase (CAT), flavonoids, carotenoids, proline, and soluble sugars, mitigate the effects of salinity-induced oxidative stress^27–30^.

On the other hand, plant tolerance to salinity stress is highly dependent on plant species and both duration and severity of the stress^31^. It has also been confirmed that genetic variations, salt tolerance degree, and plant responses can vary in plant species and different cultivars of a species^32–35^. Bread wheat (*Triticum aestivum* L.), a staple crop for more than half of the world’s population^36–38^, exhibits variable responses to salinity across germination and growth stages, emphasizing the need to identify key traits linked to salinity tolerance to improve yield and its components^39–43^. Based on current findings, the propagation of salt-tolerant inbred lines is recognized as a promising approach to mitigate the detrimental effects of salinity on plant growth and development^44,45^. In this regard, some studies have shown that salt-tolerant wheat genotypes maintain higher K⁺/Na⁺ and Ca^2+^/Na^+^ ratios under saline conditions compared to more sensitive varieties, resulting in improved growth, development, and yield-related attributes^46–48^. Additionally, evidence suggests that salt-tolerant genotypes accumulate greater levels of proline than salt-sensitive ones, although chlorophyll content tends to decline across all genotypes subjected to salt stress^49^. Therefore, a deeper understanding of plant tolerance mechanisms, such as physiological, metabolic, and biochemical responses, under salinity stress can contribute to enhancing crop yield and its components under varying levels of salt stress^50,51^.

In Iran, bread wheat accounts for approximately 67% of total agricultural production and plays a crucial role in national food security^52,53^. However, nearly half of the croplands are affected by salinity, posing a significant challenge for wheat cultivation^54,55^. Therefore, developing salt-tolerant cultivars or promising lines through breeding programs is essential to improve productivity in these susceptible areas. In the meantime, Charam (Choram) County, located in the south and west of the province and adjacent to Khuzestan, has an arid and semi-arid climate with low rainfall and rare cold weather/frost. Also, the majority of the people of this region earn a living through agricultural activities (mainly the cultivation of wheat, barley, rice, corn, beans, etc.). In general, few comprehensive studies have examined the biochemical and functional characteristics of bread wheat varieties in the study area, particularly under salinity stress. Therefore, this study aimed to compare germination-related responses of selected wheat cultivars and promising lines under different salinity levels. Furthermore, morpho-physiological, biochemical, and functional traits were evaluated to identify the most suitable cultivars for cultivation in normal and saline fields. Considering the extensive data of the studied work, the obtained results are expected to serve as a reference for other regions with similar climates.

## Material and methods

In the present study, plant responses of various cultivars and new promising lines of winter wheat (Chamran-2, Mehregan, Marvdasht, and Narin cultivars, as well as the new lines MS-89-13 and MS-90-13) were first investigated under Petri dish conditions (Table 1). Accordingly, petridish experiments were first performed to obtain adequate knowledge about seed germination indices and some growth traits under different levels of salt stress (i.e., 2 (control), 4, 8, and 12 dS·m^−1^) using a factorial design based on the completely randomized design in three replications. Subsequently, the most salt-tolerant cultivars identified from the Petri dish test were used in the field experiment, which was performed as a combined analysis based on a randomized complete block design in two experimental fields (normal and saline) located within the same research station in Charam (Choram) County (30°45′04′′N and 50°44′34′′E), Kohgiluyeh and Boyer-Ahmad Province, Iran, during two growing seasons of 2020–2021 and 2021–2022 in three replications. Prior to field experiments, soil and irrigation water samples from both the normal and saline fields (Tables 2 and 3, respectively) were analyzed in the Soil and Water Laboratory to determine their physicochemical properties, and required nutrients were applied accordingly. In addition, since detailed meteorological data were unavailable for the experimental site, the year factor was included in the statistical analysis to account for potential inter-annual variation in environmental conditions.

**Table 1:** Physicochemical properties of water samples in the two experimental regions.

Genotype/Cultivar	Pedigree	Origin	Type	Key characteristics / Adaptation
Chamran-2	Attila 50Y//Attila /Bacanora	AREEO, Iran	Bread wheat	Tolerant to terminal heat, acceptable rust resistance, good bread quality
Mehregan (S-87-20)	OASIS/ SKAUZ// 4BCN / 3 / 2PASTOR	AREEO, Iran	Bread wheat	High yield potential; resistant to yellow, leaf, and stem rusts; good adaptation to warm irrigated regions of Iran
Marvdasht	HD172 / Bloundane // Azadi	ANRRC, Iran (derived from CIMMYT germplasm)	Bread wheat	High yield; resistant to leaf rust; well adapted to southern, western, and southwestern regions of Iran
Narin (MS-87-8)	1-66-22 / 3 / Alvd // Aldan / Ias58	Improvement Institute (SPII), AREEO, Karaj, Iran	Bread wheat	Derived from a cross between a salt-tolerant local line (1-66-22) and the high-yielding cultivar Pishtaz; good adaptability to saline conditions
MS-89-13	Kauz*2/Opata//Kauz/3/Sakha 8/4/Tam 200	Seed and Plant Improvement Institute (SPII), AREEO, Karaj, Iran	Bread wheat	Promising line derived from multiple CIMMYT and Egyptian crosses; good yield potential and disease tolerance
MS-90-13	Pishtaz // Karchia	Seed and Plant Improvement Institute (SPII), AREEO, Karaj, Iran	Bread wheat	Promising line with potential tolerance to abiotic stresses and good adaptability

AREEO. Agricultural Research Education and Extension Organization.

ANRRC. Agricultural and Natural Resources Research and Education Center.

CIMMYT. International Maize and Wheat Improvement Center.

/. Indicates a cross between two parents.

//. Indicates a second cross, where the progeny of the first cross is crossed with another parent.

* Kauz was used twice in the crossing scheme before combining with Opata.

**Table 2:** Physicochemical properties of irrigation water under different salinity conditions (Normal and Saline).

Water samples	EC (dS·m^−1^)	SAR	pH	Bicarbonate (meq·L^−1^)	Cl^−^ (meq·L^−1^)	Ca^2+^ (meq·L^−1^)	Mg^2+^ (meq·L^−1^)	Na^−^ (meq·L^−1^)
Normal sample	0.007	0.2	7.47	4.9	1.4	2.4	10.9	2.1
Saline sample	8.03	14.7	9.8	13.3	48.6	28.1	21.7	51.9

**Table 3:** Soil physicochemical properties of experimental fields under normal and saline conditions.

Experimental farms	Soil texture	pH	Ec (dS ·m^−1^)	Organic carbon (%)	Available nitrogen (%)	P (mg·kg^−1^)	K (mg·kg^−1^)	Cu (ppm)	Fe (ppm)	Zn (ppm)
Normal field	Clay Loam	7.5	1.52	1.03	0.12	23.4	143.8	2.4	27.4	0.9
Saline field	Clay Loam	9.1	5.05	0.21	0.009	47. 5	231.1	5.8	43.2	1.7

Both fields were located in the same research station but differed in soil and irrigation water salinity.

At the end of the petri test, indices of germination percentage (GP), mean time to germination (MTG), speed of germination (SG), time to reach 50% of final/maximum germination (T_50_), shoot length (SL), root length (RL), root length stress tolerance index (RLSI), leaf proline content (LPC), and leaf protein (LPr) were investigated in petridish cultures. After selecting the superior wheat cultivars, field experiments were conducted on plots measuring 12 m^2^ (3 m × 4 m), each consisting of 16 rows with 20 cm row spacing. Land preparation included discing and rolling operations before planting. Furthermore, indices of the plant height (PH), 1000-grain weight (TGW), grain yield (GY), biological yield (BY), straw yield (SY), content of chlorophyll α (Chl α), chlorophyll b (Chl b), total chlorophyll (T Chl), carotenoids (CAR), leaf proline content (LPC), catalase activity (CAT), superoxid dismutase (SOD), soluble sugars (SS), grain protein (GPr), wet gluten content (GC), and gluten index (GI) were separately investigated for each field experiments based on following equations. It should be noted that biochemical and physiological analyses were conducted at specific growth stages according to the BBCH scale (Table 4). Photosynthetic pigments (chlorophyll α, chlorophyll b, total chlorophyll, and carotenoids), leaf proline content, soluble sugars, and enzymatic activities (catalase, superoxide dismutase) were measured during the flowering stage (BBCH 60-69), when the leaves are fully expanded and metabolic activity is maximal. Grain protein content, wet gluten, and gluten index were analyzed at physiological maturity (BBCH 90-99) to ensure maximal accumulation in the grains^56^.

**Table 4:** Growth stages of wheat (BBCH scale) for biochemical and physiological analyses.

Parameter / Trait	Growth stage (Phenophase)	BBCH code	Description
Photosynthetic pigments (Chl a, Chl b, T Chl, and CARs)	60–69	Flowering	Full flower opening; leaves fully expanded; peak metabolic activity. Carotenoids are included as photosynthetic pigments with a protective role against oxidative stress
Non-enzymatic antioxidants (Leaf proline content and soluble sugars)	60–69	Flowering	
Enzymatic activities (CAT and SOD)	60–69	Flowering	
Grain protein content, wet gluten, and gluten index	90–99	Physiological maturity	Fully mature grain; maximum accumulation of storage compounds (protein and gluten)

### Germination indices

#### Germination percentage

Twenty-five sterilized seeds were first placed into 25 × 150 mm Petri dishes lined with a single filter paper soaked with the studied treatments (i.e., 2, 4, 8, and 12 dS·m^−1^) in three replicates. The germinated seeds were then counted after ten days to estimate the germination percentage (GP) as follows^57^:

1 $${\mathrm{GP}} (\% ) = \frac{n}{N} \times 100$$

where n and N refer to the total number of germinated seeds and the total number of seeds, respectively.

#### Mean time to germination (MTG)

The mean time to germination (MGT) index is defined as a measurement of the rate/time spread of germination and is shown as an average time assumed for seeds to germinate, which is usually calculated by the following equation ^58^:

2 $$\text{MTG }\mathrm{=}\frac{\left(\mathrm{N}1\mathrm{T}1\right)+ \left(\mathrm{N}2\mathrm{T}2\right)+\dots +\left(\mathrm{NiTi}\right)}{n}$$

In which N, T, and n refer to the number of seeds germinated on day x, the time from the beginning of the germination test in terms of days, and the number of newly germinated seeds.

#### Speed of germination (SG)

Speed of germination (germination rate) is estimated by dividing the number of normal seedlings tagged each day by the number of days in which seeds were tagged into a seed germinator. Then, the SG parameter was calculated according to Eq. 3, in which higher values indicate greater and faster germination and vice versa^57^.

3 $$\text{SG } = \frac{n1}{d1}\text{ + }\frac{n2}{d2}+ \frac{n3}{d3}+ \frac{ni}{di}$$

where “n” is the number of germinated seeds and “d” is the number of days.

#### Root length stress tolerance index (RLSI)

After ten days, the test was completed, and the stem and root lengths of the studied seedlings were recorded as the stem length stress tolerance index and root length stress tolerance index, as follows^59^:

4 $$\text{RLSI (\%) =}\frac{\text{root length of stressed plants}}{\text{root length of control plants}}\times 100$$

#### Leaf proline content (LPC)

Proline measurement was performed according to the Bates et al.^60^ method. Accordingly, 0.5 g of fresh tissue of the leaf blade for each sample was first weighed and rubbed in 10 ml of 3% aqueous sulfosalicylic acid, and the homogenate was filtered through Whatman filter paper. Then, 2 ml of acid-ninhydrin and 2 ml of glacial acetic acid were added to 2 ml of the filtrated extract and reacted for 60 min at 100 °C. In the next step, test tubes were placed in an ice bath for 30 min. Then, 4 ml of toluene was added to the reaction mixture, mixed vigorously with a test tube stirrer for 15–20 s, and kept at room temperature for a short time. At this stage, two separate layers were created, and the upper phase was separated. Next, the absorbance was read at 520 nm using a spectrophotometer. Eventually, the proline concentration was determined from the standard curve (at concentrations of 0, 50, 100, 200, and 250 µM) and calculated based on the µM·g^−1^ fresh weight (Eq. 5).

5 $$Leaf\; Proline\; Content \left( {\mu {\mathrm{M}} \cdot g^{ - 1} FW} \right) = \frac{{\left[ {\frac{{\left( {\mu {\mathrm{g}}.{\mathrm{ml}}^{ - 1} {\text{ proline}} \times {\text{ ml toluene}}} \right)}}{{115.5{ }\mu {\mathrm{g}} \cdot \mu {\mathrm{mole}}^{ - 1} }}} \right]}}{{\left[ {\frac{{\left( {\text{g sample}} \right)}}{5}} \right]}}$$

#### Photosynthetic pigments (including chlorophyll a, chlorophyll b, total chlorophyll, and carotenoids)

Chlorophyll a (Chl a), chlorophyll b (Chl b), total chlorophyll (Chl T), and carotenoids (CAR) were calculated using methods presented by Arnon^61^ and Lichtenthaler^62^. Accordingly, 0.5 g of fresh tissue of the leaf blade for each sample was first weighed and rubbed in a stone mortar with 80% aqueous acetone. Then, the obtained scum was isolated by a filter paper and a volumetric flask, and the resulting scum was again rubbed and then isolated. Next, the product was diluted to 10 ml with 80% acetone and immediately transferred to a cell and absorbed by a spectrophotometer (Jenway 6300 model) at different wavelengths of 645, 663, and 470 nm. Eventually, Chl a, b, total Chl, and CAR contents were determined using the following formulas. It should be noted that 80% aqueous acetone was used as a Blank solution.

6 $$Chlorophyll\; a\; (Chla)=\frac{[\left(12.7\times D663\right)-\left(2.69\times D645\right)]\times V}{1000\times W}$$

7 $$Chlorophyll\; b\; (Chlb)=\frac{[\left(22.9\times D645\right)-\left(4.93\times D663\right)]\times V}{1000\times W}$$

8 $$Total\; Chlorophyll\; (ChlT)=\frac{[\left(20.2\times D645\right)-\left(8.02\times D663\right)]\times V}{1000\times W}$$

9 $$Carotenoids\; (CAR)=\frac{[\left(1000\times D470)-(1.82\times Chl.a)-(85.02\times Chl.b\right)]}{198}$$

In which V is the final volume of extract per milliliter, W is tissue weight per gram, and D is optical absorption.

### Enzymatic activities of catalase(CAT) and superoxide dismutase (SOD)

Enzyme activities of CAT and SOD were respectively evaluated according to the methods described by Bailly et al.^63^ and Giannopolitis & Ries^64^ with minor modifications. In general, leaf tissues (1 g) were ground in a mortar containing 3 mm of 50 mM phosphate buffer (pH = 7.2), 1 mM ethylenediamine tetraacetic acid (EDTA), 1 mM Phenyl Methanesulphonyl Fluoride (PMSF), and 1% polyvinylpyrrolidone (PVP) to prepare enzyme extract samples. The homogenate was centrifuged at rpm = 14,000 g for 15 min at 4 °C, and the supernatant was used to measure the activity of the CAT enzyme. Finally, the catalase activity was determined based on the rate of H_2_O_2_ disappearance at 240 nm wavelength with the reaction mixture containing 25 mM potassium phosphate buffer (pH 7.0), 10 mM H_2_O_2_, and enzyme extract (ε = 0.036 M^−1^ cm^−1^).

To assay the superoxide dismutase (SOD) activity, fresh leaves (1 g) were also homogenized in a reaction mixture containing 50 mM phosphate buffer, 0.013 mM methionine, 0.1 μm EDTA, and 2 μm riboflavin, which was kept in full darkness. Immediately after adding the riboflavin, 3 ml of the obtained solution was poured into the test tube, and 100 ml of the protein extract was added to each sample. Test tubes were placed 30 cm from the light source for 16 min, and the spectrophotometer was set. Then, samples were read at 560 nm with a blank solution as a control treatment. Finally, the SOD enzyme activity was calculated based on the enzyme units per mg of protein for all samples.

#### Soluble sugars (SS)

Soluble sugars were evaluated using the method described by Yemm and Willis^65^ with slight modifications. Briefly, 0.1 g of the dry leaves was weighed and hydrolyzed in boiling water for three hours in the presence of 5 ml of 2.5 N HCl. In the next step, the samples were cooled at room temperature and neutralized with solid sodium carbonate until effervescence ceased. Then, the obtained solutions were diluted to 100 mL with distilled water and centrifuged at 3500 rpm for 10 min. Then, 0.5 ml of the supernatant was collected and diluted to 1 ml with distilled water. In the next step, 4 mL of anthrone reagent was added to each tube and placed in a boiling water bath for eight minutes. Next, the absorbance of each sample was read at 630 nm, and a glucose-free tube was considered for the control solution. Eventually, the soluble sugar contents were expressed in µg·g^−1^ DW.

#### Grain protein (GPr)

The crude protein content in the defatted flour was analyzed by the Kjeldahl method as described by Jones^66^ as follows:

10 $$Seed\; protein (SP)=\mathrm{N\%}\times 6.25$$

where N is considered as the percentage of nitrogen obtained by the Kjeldahl method.

#### Wet gluten (WG) and gluten index (GI)

The mentioned parameters were estimated according to Equations cited by Hussain et al.^67^ and Popa et al.^68^, respectively.

11 $$\mathrm{Wet}\; \text{Gluten }(\mathrm{\%})=\frac{\text{Total wet gluten }\left(\mathrm{g}\right)\times 860}{100-{\% sample moisture}}$$

12 $$\text{WGS }(\mathrm{g})=\frac{\text{GI }}{100}\times \text{WG }(\mathrm{\%})$$

where WGS, GI, and WG referred to the wet gluten remaining on the sieve (g) at centrifugation, gluten index, and wet gluten (%). Furthermore, it is stated that GI < 30%, 30 ≤ GI ≤ 80%, and GI > 80% are wheat cultivars with weak, normal, and strong quality degrees of gluten, respectively^42^.

### Statistical analysis

The data were analyzed by SAS software (version 9.4), and the LSD test was used at *p* < 0.05 to compare the means of the data for two petri dishes and field experiments. Also, the charts were drawn using Microsoft Excel software. The Bartlett’s test was also performed on the homogeneity of variances in the field experiments.

## Results

### Petri culture experiments

#### Germination indices

Results of the ANOVA (Table 5) demonstrated that all studied indices of GP, MTG, SG, T_50_, SL, RL, RISI, LP, and LPr affected by salinity levels at *p* < 0.01 while cultivar treatment had significant effects on GP, and T_50_ at *p* < 0.05 and on SL, RL, LP, and LPr indices at *p* < 0.01. However, the cultivar treatment had no significant effects on MTG and SG indices. In addition, results indicated that the RLSI trait was affected by the interaction of cultivar × salinity levels at *p* < 0.01 (Table 5).

**Table 5:** Combined analysis of variance of germination indices of wheat cultivars (mean square) in petri culture.

Source of variation	df	GP	MTG	SG	T_50_	SL	RL	RLSI	LPC	LPr
Cultivar	5	69.73 *	0.24 ns	1.53 ns	0.55 *	4.41 **	9.16 **	975.89 **	3.37 **	0.05 **
Salinity	3	1574.15**	3.09 **	61.29 **	13.54 **	170.22 **	162.88 **	18,131.02 **	60.39 **	0.39 **
Cultivar × Salinity	15	16.64 ns	0.05 ns	0.85 ns	0.14 ns	0.58 ns	1.88 **	204.62 **	0.29 ns	0.007 ns
Error (E)	48	21.11	0.19	0.84	0.21	0.63	0.49	60.68	0.55	0.009
CV (%)		5.1	10.11	11.59	11.47	13.48	11.47	12.05	14.04	9.57

ns: non-significant; ** and *: significant at *p* < 0.01 and *p* < 0.05, respectively.

The mean comparisons of cultivar and salinity level effects on germination indices and selected growth traits are presented in Table 4. The data show that the highest germination percentage (GP) was recorded for the Chamran-2 cultivar, reaching 93.33%, which was significantly higher than those of MS-89-13 and MS-90-13. In contrast, the lowest GP (87.33%) was observed for the MS-90-13 line. Overall, Chamran-2 demonstrated notable improvements in GP, with increases of 1.08%, 3.70%, 3.32%, 6.46%, and 6.87% compared to Mehregan, Marvdasht, Narin, MS-89-13, and MS-90-13, respectively. Additionally, the control treatment consistently produced higher GP values than the salinity levels of 4, 8, and 12 dS·m^−1^, with respective reductions of 4.65%, 16.58%, and 42.41% in GP (Table 6).

**Table 6:** Mean comparison (mean square) of the investigated germination indices.

Treatments	GP (%)	T_50_ (Seedlings·day ^−1^)	SL (cm)	LPC (mg·g^−1^ FW)	LPr (mg·g^−1^ FW)
*Cultivars*					
Chamran-2	93.33^a^	3.61^b^	6.94^a^	4.55^d^	1.06^a^
Mehregan	92.33^a^	4.18^a^	6.1^b^	4.99^cd^	0.98^bc^
Marvdasht	90^ab^	3.97^ab^	5.48^bc^	5.75^ab^	0.94^cd^
Narin	90.33^ab^	4.13^a^	5.86^bc^	6.02^a^	1.03^ab^
MS-89–13	87.67^b^	4.13^a^	5.25^c^	5.25^bc^	0.91^cd^
MS-90–13	87.33^b^	4.13^a^	5.56^bc^	5.16^bc^	0.89^d^
LSD	3.77	0.38	0.65	0.61	0.08
*Salinity levels (dS*·*m*^−*1*^*)*					
2 (Control)	100^a^	3.02^c^	9.07^a^	3.36^d^	1.13^a^
4	95.56^b^	3.86^b^	7.78^b^	4.33^c^	1.04^b^
8	85.78^c^	4.07^b^	4.09^c^	5.91^b^	0.92^c^
12	79.33^d^	5.13^a^	2.51^d^	7.54^a^	0.72^d^
LSD	3.08	0.31	0.53	0.5	0.06

Means sharing the same superscript are not significantly different from each other at *p* < 0.05.

**Fig. 1:**
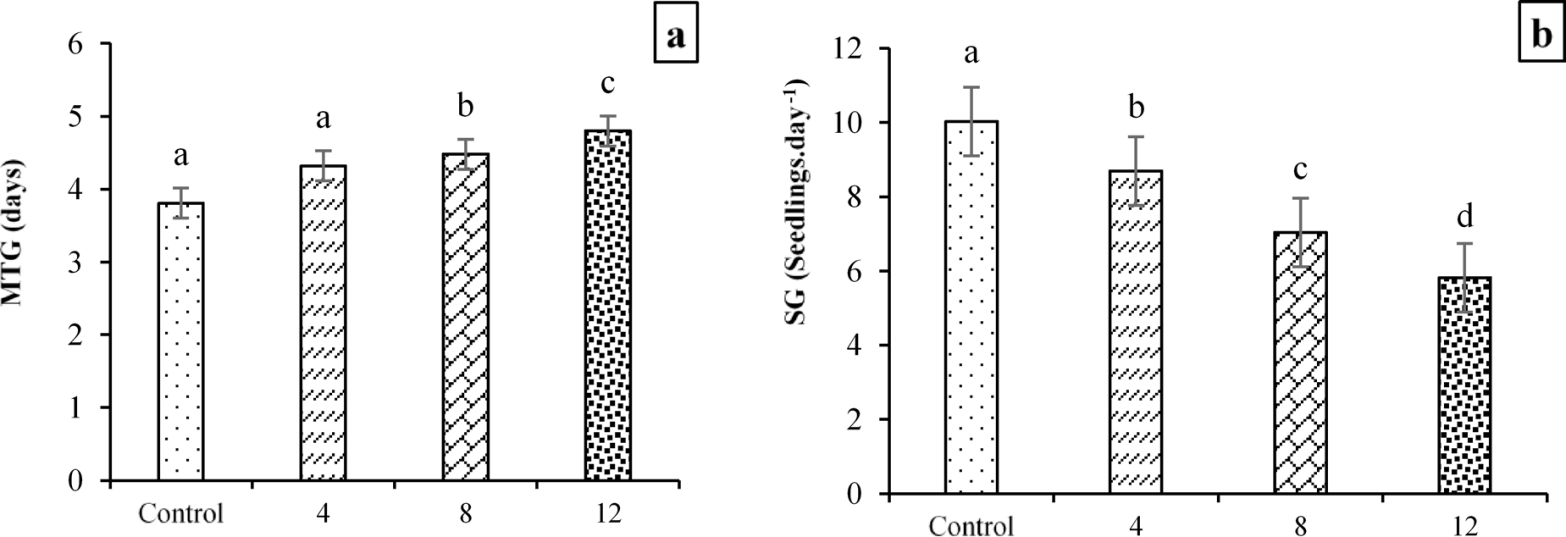
Effects of salinity levels (mean square) on MTG (**a**) and SG (**b**) for different bread wheat cultivars. Means sharing the same superscript are not significantly different from each other at *p* < 0.05.

The effects of different salinity levels on mean time to germination (MTG) and seedling growth rate (SG) are illustrated in Fig. 1a and b, respectively. As shown in Fig. 1a, the shortest MTG (3.81 days) was recorded under the control treatment (2 dS·m^−1^), while the longest MTG (4.80 days) occurred at 12 dS·m^−1^ salinity. In other words, seeds exposed to 12 dS·m^−1^ required more time to germinate, with MTG values increasing by 25.98%, 11.11%, and 7.14% compared to those under 2, 4, and 8 dS·m^−1^ salinity, respectively. In contrast, the highest SG value (10.03 seedlings·day^−1^) was observed under the 2 dS·m^−1^ treatment, which represented significant increases of 15.45%, 42.4%, and 72.3% relative to the 4, 8, and 12 dS·m^−1^ salinity levels, respectively (Fig. 1b).

The average values of simple effects of cultivar treatment on T_50_ (Table 6) showed that the highest T_50_ (with a value of 4.18 seedlings·day^−1^) was recorded for Mehregan cultivar while had no significant difference with T_50_ value in Narin cultivar (with a value of 4.18 seedlings·day^−1^), and the lowest T_50_ value (equivalent to 3.61 seedlings·day^−1^) was obtained for the Chamran-2 cultivar. Statistical calculations of the T_50_ data indicated increases of 15.79, 5.29, 1.21, 1.21, and 1.21% in the Mehregan cultivar compared to Chamran-2, Marvdasht, Narin, MS-89-13, and MS-90-13, respectively. Furthermore, the highest values for SL and RL traits (6.94 and 7.67 cm) were observed for the Chamran-2 cultivar, while their lowest values (equal to 5.25 and 5.22 cm) were achieved for the MS-89-13 cultivar. In general, the Chamran-2 cultivar compared to cultivars of Marvdasht, Mehregan, Narin, MS-89-13, and MS-90-13 had increases of 13.77, 26.64, 18.43, 32.19, and 24.82% for the SL trait and equal to 17.82, 33.86, 29.34, 46.93, and 36.72% for the RL index, respectively. In relation to differences between contents of LPC and LPr of six cultivars in the present study, results showed that maximum and minimum of LPC (with values of 6.02 and 4.55 mg·g^−1^ FW) were estimated for Narin and Chamran-2 coltivars, and maximum and minimum content of LPr (with values of 1.06 and 0.89 mg·g^−1^ FW) were measured for Chamran-2 and MS-90-33 coltivars, exclusively. In general, evidence explained that the LPC in the Narin cultivar increased significantly by 32.31, 20.64, 4.7, 14.67, and 16.67% compared to other cultivars of Chamran-2, Mehregan, Marvdasht, MS-89-33, and MS-90-33. Also, Chamran-2 had significant increases of 8.16, 12.77, 6, 16.48, and 19.1% in the LPr index than the Mehregan, Marvdasht, Narin, MS-89-33, and MS-90-33 cultivars, respectively (Table 6).

In addition to the above, data related to the effects of different levels of salinity stress on the studied traits illustrated that the GP value for salinity level of 2 dS·m^−1^ (control treatment; GP = 100%) was significantly higher than salinity levels of 4, 8, and 12 dS·m^−1^, equal to 4.64, 16.58, and 26.06%, respectively. Furthermore, the maximum value for T_50_ (4.95 seedlings·day^−1^) was achieved for salinity levels of 12 dS·m-1, which had increases of 69.87, 32.9, and 26.04% compared to the 2, 4, and 8 salinity treatments. Since salinity levels had negative concentration-dependent effects on germination indices, SL trait reciprocally decreased under the application of 12 dS·m^−1^ salinity equal to 72.33, 67.74, and 38.63% compared to the levels of 2, 4, and 8 dS·m^−1^ (Table 6).

**Fig. 2:**
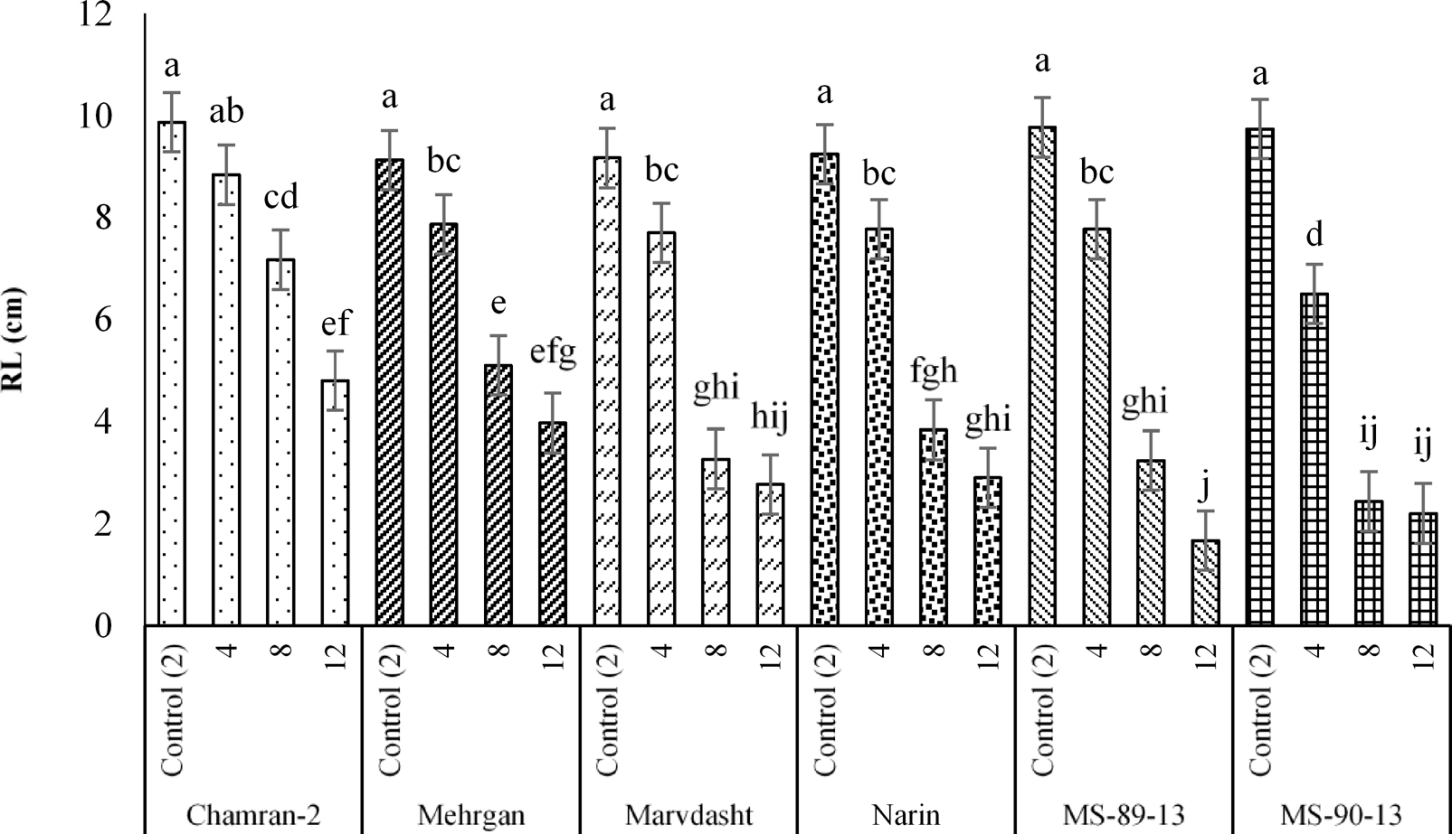
Interaction effects of cultivar × salinity levels (mean square) on the RL trait of different bread wheat cultivars. Means sharing the same superscript are not significantly different from each other at *p* < 0.05.

**Fig. 3:**
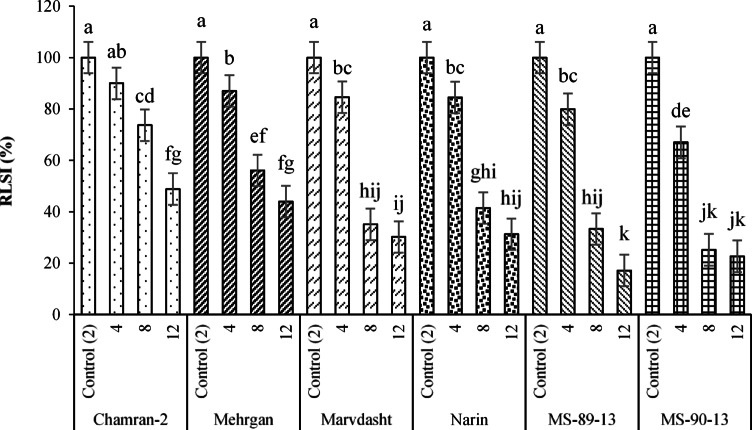
Interaction effects of cultivar × salinity levels (mean square) on RLSI trait of different bread wheat cultivars. Means sharing the same superscript are not significantly different from each other at *p* < 0.05.

Plus, since the RL and RLSI traits were affected by the interaction of cultivar × salinity levels, the corresponding outputs are shown in Figs. 2 and 3. Based on the data, the maximum and minimum RL (with equivalent averages of 9.87 and 1.67 cm) were acquired for the interactions of Chamran-2 × control (2 dS·m^−1^) and MS-90-33 × 12 dS·m^−1^ (Fig. 2). The lowest value for RLSI (equal to 17.13%) was also estimated for the interaction of MS-90-33 × 12 dS·m^−1^ (Fig. 3).

Furthermore, the results indicated that the highest levels of leaf proline content (LPC) and leaf protein (LPr) were recorded under the 12 dS·m^−1^ and control (2 dS·m^−1^) treatments, respectively, with values of 7.57 and 1.06 mg·g^−1^ FW. In contrast, the lowest LPC (3.50 mg·g^−1^ FW) was observed under the control treatment, while the lowest LPr content (0.84 mg·g^−1^ FW) occurred at 12 dS·m^−1^ salinity. Compared to the control, LPC increased by 124.41%, 74.13%, and 27.58% under 12 dS·m^−1^ in comparison with 2, 4, and 8 dS·m^−1^ salinity levels, respectively. Conversely, LPr content significantly decreased by 36.28%, 30.77%, and 21.74% under 12 dS·m^−1^ relative to the same salinity levels (Table 4). Based on the germination indices and salinity tolerance observed in the Petri dish experiment, four cultivars were selected as the most promising candidates and were further evaluated under field conditions for more comprehensive analysis.

### Field experiment

#### Morphological and yield-related indices

The ANOVA results (Table 7) showed that the PH trait was affected by field conditions, year, and cultivar at *p* < 0.05. However, the interactions of field conditions × year, field conditions × cultivar, year × cultivar, and field conditions × year × cultivar had no significant effects on this trait. Moreover, despite the notable effects of field conditions, year, and cultivar treatments on TGW (*p* < 0.01) and on the GY trait (at *p* < 0.01, *p* < 0.05, and *p* < 0.01, respectively), none of the interaction effects showed statistically significant differences for these traits. Also, the SY trait was affected by treatments of field conditions, year, and cultivar, and cultivar × field conditions × year interaction at *p* < 0.01, while the BY index was affected by field conditions and cultivar treatments at *p* < 0.01 (Table 7).

**Table 7:** Combined analysis of variance (mean square) for morphological and yield-related attributes od different bread wheat cultivars.

S.O.V	df	PH	TGW	GY	SY	BY
Block	8	14.77 ns	45.27 ns	302,708.42 ns	514,941.34 ns	857,751.5 ns
Field conditions	1	281.79*	390.62**	44,456,316.45**	12,789,870.88**	104,051,192.4**
Year	1	231.00*	241.70**	2,401,504.53*	5,047,049.83**	427,171.9 ns
Field conditions × Year	1	59.63 ns	59.34 ns	260,404.31 ns	385,603.32 ns	1,183,671.4 ns
Cultivar	3	154.44*	1650.64**	4,678,500.39**	3,581,589.41**	16,546,608.1**
Cultivar × Field conditions	3	7.67 ns	21.39 ns	229,155.26 ns	255,751.06 ns	926,762.5 ns
Cultivar × Year	3	16.47 ns	2.02 ns	430,049.84 ns	64,840.82 ns	797,603.2 ns
Cultivar × Field conditions × Year	3	3.40 ns	3.61 ns	98,192.02 ns	1,261,085.87**	1,432,752.7 ns
Error (E)	24	45.25	26.63	498,730.48	271,525.13	878,768.9
CV (%)		7.90	12.79	16.22	10.42	10.01

ns: non-significant; ** and *: significant at *p* < 0.01 and *p* < 0.05, respectively.

According to the mean comparisons data (Table 8), it was observed that the PH trait of plants grown in the normal field (normal canditions) was significantly higher (equal to 5.8%) than that of wheat plants grown in the saline field. Also, wheat plants in the second year had higher heights, equivalent to 5.28% more than plants cultivated in the first year. Among different cultivars, the results revealed that the highest and lowest significant values for the PH trait were obtained respectively for cultivars of Chamran-2 and Narin, with equivalent values of 89.59 and 81.91 cm. Statistical calculations in Table 6 showed that the PH index in the Chamran-2 cultivar compared to Mehregan, Marvdasht, and Narin cultivars had a significant increase of 3.54, 8.45, and 9.38%, respectively. In addition, data relevant to the TGW index showed that wheat plants cultivated in the normal field had higher increases than plants in saline conditions, and plants farmed in the second year had increases equal to 15.2 and 11.78%, respectively. However, Chamran-2 cultivar (with an average TGW equal to 57.44 g), in comparison with cultivars of Mehregan (38.29 g), Marvdash (34.27 g), and Narin (31.44 g), produced seeds with heavier TGW equal to 50.01, 67.61, and 82.7%. Concerning the effects of different treatments on GY trait, the results illustrated that the highest GY was achieved under treatments of the normal farm, the second year data, and the Chamran-2 cultivar. In general, it was assessed GY improved in the normal field than in the saline conditions (equal to 56.77%), the second year compared to the first year (equal to 10.83%), and the Chamran-2 cultivar than the cultivars of Mehregan, Marvdasht, and Narin with equivalent values of 15.41, 38.09, and 28.78%, respectively (Table 8).

**Table 8:** Mean comparison (mean square) of PH, TGW, GY, BY, Chl b, SP, GC, and GI traits under the application of main treatments of field conditions, year, and cultivar.

Treatments	PH (cm)	TGW (g)	GY (kg·ha^−1^)	BY (kg·ha^−1^)	Chl b (mg·g^−1^ FW)	GPr (%)	WG (g)	GI (%)
*Field conditions*								
Normal conditions (Normal field)	87.58^a^	43.21^a^	5315.3^a^	10,833.4^a^	0.93^a^	11.45^a^	34.34^a^	32.21^a^
Saline conditions (Saline field)	82.74^b^	37.51^b^	3390.6^b^	7888.8^b^	0.78^b^	10.19^b^	27.42^b^	27.68^ab^
*Year*								
First year	82.97^b^	38.12^b^	4129.3^b^	9455.4^a^	0.74^b^	11.08^a^	29.45^b^	28.76^a^
Second year	87.35^a^	42.61^a^	4576.6^a^	9266.8^a^	0.97^a^	10.56^a^	32.31^a^	31.14^a^
LSD	4.01	3.07	420.76	558.52	0.14	0.72	2.04	2.57
*Genotype*								
Chamran-2	89.59^a^	57.44^a^	5171^a^	10,816.3^a^	1.07^a^	11.51^a^	32.48^ab^	30.13^ab^
Mehregan	86.53^b^	38.29^b^	4480.7^b^	9765.5^b^	0.86^b^	11.17^ab^	33.46^a^	32.54^a^
Marvdasht	82.61^b^	34.27^bc^	3744.7^c^	8180^c^	0.76^b^	10.26^b^	27.68^c^	26.73^b^
Narin	81.91^b^	31.44^c^	4015.3^bc^	8682.5^c^	0.74^b^	10.35^b^	29.90^bc^	30.40^a^
LSD	5.67	4.35	595.04	789.86	0.19	2.06	2.89	3.64

Means sharing the same superscript are not significantly different from each other at *p* < 0.05.

**Fig. 4:**
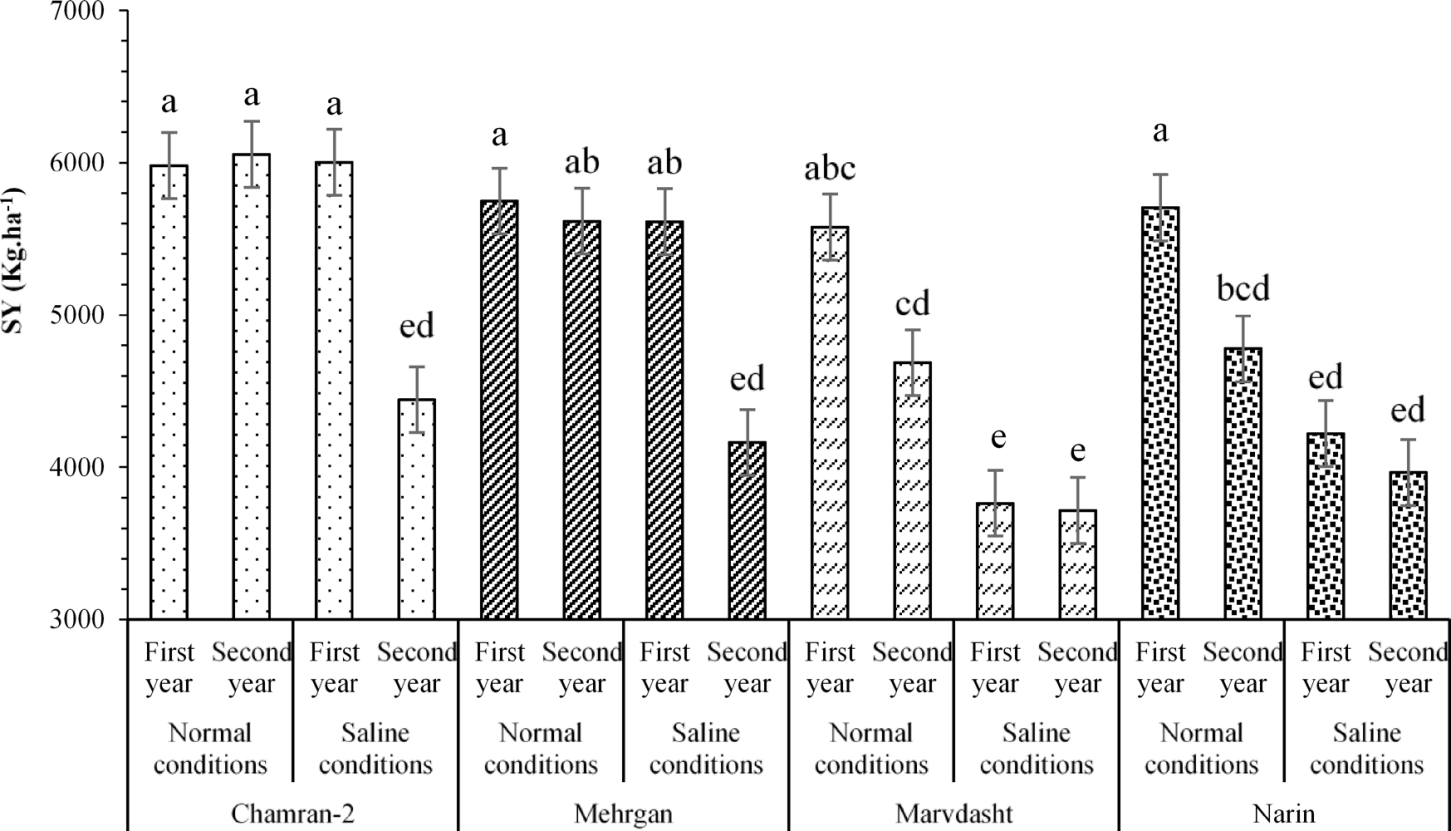
Effects of triple interaction of field conditions × year × cultivar (mean square) on wheat straw yield. Means sharing the same superscript are not significantly different from each other at *p* < 0.05.

Effects of the three-way interaction of field conditions × year × cultivar on wheat straw yield are shown in Fig. 4. As can be seen, the results indicated that maximum and minimum values for the SY trait were achieved under interactions of Chamran-2 cultivar × normal field × second year and Marvdasht cultivar × saline conditions × second year with amounts of 6054.06 and 3715.75 kg.ha^−1^.

According to data (Table 8), the obtained results revealed that the normal field had a significant increase equal to 37.33% in BY trait compared to the saline conditions. On the other hand, although there was no significant difference between BY values in the two experiment years, the first year (with 9455.4 kh.ha^−1^) had an increase equal to 2.04% to the BY trait in the second year (with 9266.8 kh.ha^−1^). The highest amount of BY was achieved for the Chamran-2 cultivar with an average value of 10,816.3 kg·ha^−1^, which had a notable increase compared to the BY trait in other studied cultivars. In general, the BY trait of the Chamran-2 cultivar had significant increases equal to 10.76, 32.23, and 24.58% compared to BY in cultivars of Mehregan, Marvdasht, and Narin, respectively (Table 8).

### Biochemical indices

#### Photosynthetic pigments (Chl α, Chl b, T Chl, and CAR)

The results of Table 5 indicated that Chl α affected respectively by field conditions, year, cultivar, and field conditions × year interaction at 1%, 5%, 5%, and 1% probability levels and Chl b by treatments of field conditions, year, and cultivar at 5%, 1%, and 1% probability levels. As well, treatments of field conditions, year, and cultivar at *p* < 0.01 and interactions of field conditions × year and cultivar × year at *p* < 0.05 had significant effects on T Chl content. Furthermore, the results revealed that notable differences were observed in the CAR content at *p* < 0.01 under field conditions, cultivar, and field conditions × cultivar interaction and at *p* < 0.05 for the year treatment (Table 9).

**Table 9:** Combined analysis of variance (mean square) for some biochemical traits of different bread wheat cultivars.

S.O.V	df	Chl a	Chl b	T Chl	CAR	CAT	SOD	PC	SS	GPr	WG	GI
Block	8	1.05 ns	0.14*	1.10 ns	0.15 ns	3.61 ns	51.45 ns	0.88 ns	165.68 ns	2.24 ns	3.74 ns	15.44 ns
Field conditions	1	18.90**	0.28*	23.83**	7.46**	620.07**	7474.27**	18.75**	4090.37**	18.75**	574.78**	246.16**
Year	1	6.54*	0.62**	11.16**	0.78*	147.35*	454.05**	0.51 ns	1752.33**	3.31 ns	98.33**	67.92 ns
Field conditions × Year	1	6.26*	0.07 ns	5.04*	0.35 ns	0.35 ns	0.01 ns	0.78 ns	714.87*	2.80 ns	2.21 ns	0.0001 ns
Cultivar	3	8.64**	0.30**	11.73**	2.20**	43.65 ns	263.74**	6.28*	1980.69**	4.52*	81.70**	69.17*
Cultivar × Field conditions	3	1.86 ns	0.02 ns	1.95 ns	0.93**	26.71 ns	87.95 ns	0.48 ns	121.22 ns	0.24 ns	1.47 ns	18.48 ns
Cultivar × Year	3	1.89 ns	0.05 ns	2.28*	0.10 ns	5.69 ns	22.22 ns	1.51 ns	71.23 ns	0.07 ns	2.01 ns	1.41 ns
Cultivar × Field conditions × Year	3	0.41 ns	0.03 ns	0.32 ns	0.03 ns	8.85 ns	302.43**	2.87 ns	110.71 ns	0.97 ns	3.91 ns	7.75 ns
Error (E)	24	0.87	0.05	0.82	0.13	22.32	49.1	1.35	173.56	1.46	11.74	18.67
CV (%)		12.04	26.62	10.56	12.60	11.65	10.88	10.79	11.06	11.18	11.10	14.43

ns: non-significant; ** and *: significant at *p* < 0.01 and *p* < 0.05, respectively.

**Fig. 5:**
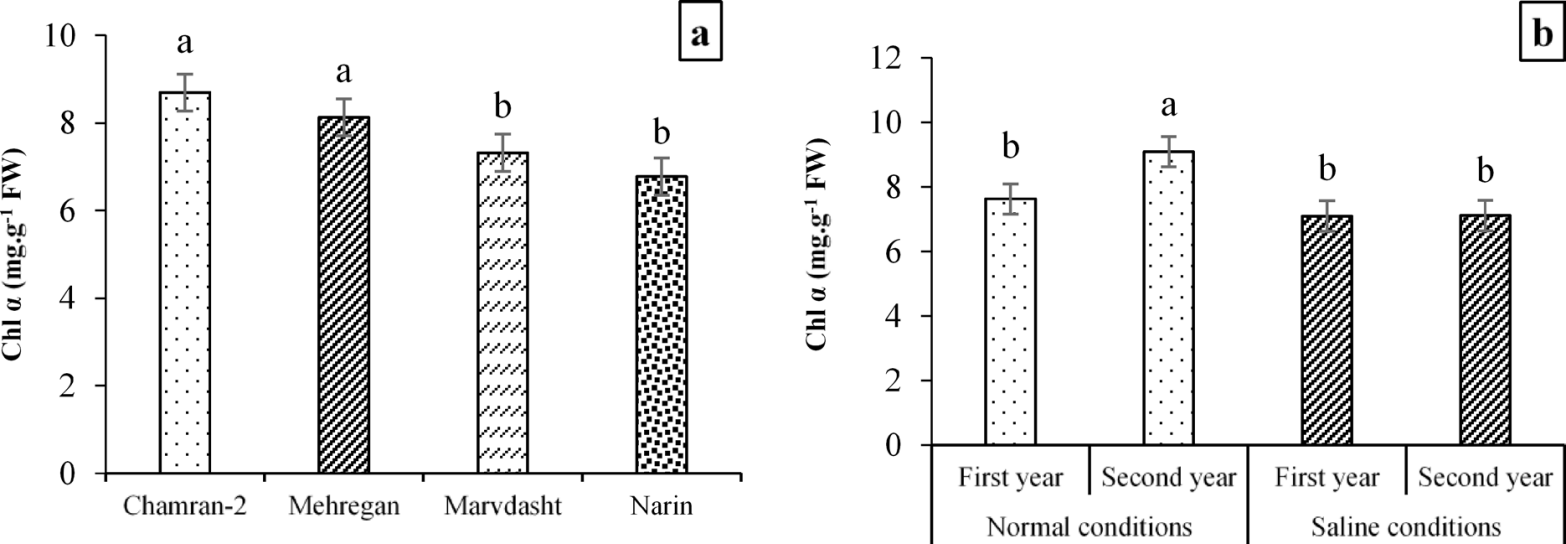
Evaluating Chl α content (mean square) under the application of different cultivars (**a**) and field conditions × year interaction (**b**). Means sharing the same superscript are not significantly different from each other at *p* < 0.05.

As can be seen, the content of Chl α affected by different cultivars, so that the highest value (equal to 8.69 mg·g^−1^ FW) was obtained under the application of Chamran-2 cultivar, and the least content with an average of 6.78 mg·g^−1^ FW was recorded for the Narin cultivar (Fig. 5a). Plus, Fig. 3b data demonstrated a significant increase in the content of Chl α under Normal field × second year of examining (9.09 mg.g^−1^ FW) compared to the content of Chl α under other interactions (Fig. 5b).

Regarding the effects of treatments on photosynthetic pigments, the results (Table 8) determined that there were significant increases in the Normal field compared to the saline conditions (equivalent to 19.23%) and the second year compared to the first year (equivalent to 31.08%) in Chl b content. Additionally, it was observed that Chl b content had significant increases equal to 12, 15, and 23% in the Chamran-2 cultivar compared to Mehregan, Marvdasht, and Narin cultivars, respectively (Table 8).

**Fig. 6:**
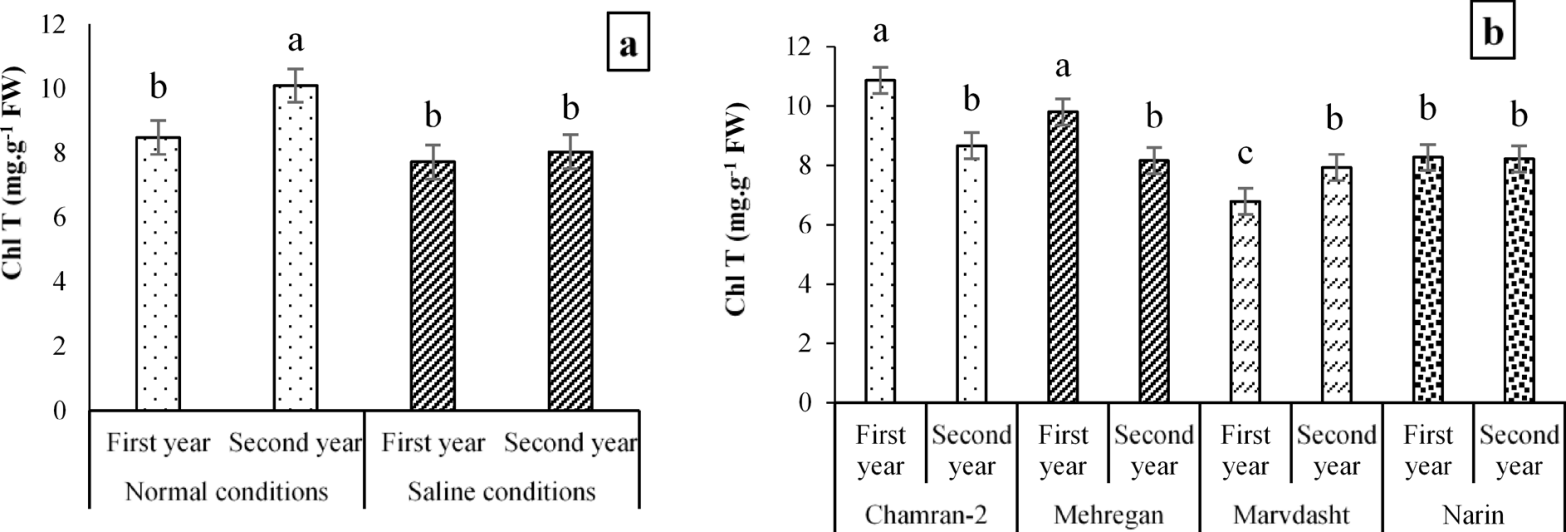
Evaluating T Chl content (mean square) under interactions of field conditions × year (**a**) and cultivar × year (**b**). Means sharing the same superscript are not significantly different from each other at *p* < 0.05.

Means comparison of field conditions × year interaction on T Chl (Fig. 6a) showed that the highest significant value and lowest value for TChl (equal to 10.10 and 7.72 mg·g^−1^ FW) were obtained for interactions of Normal field × 2nd year and saline conditions × 1st year (which no observed significant differences compared to Normal field × 1st-year and saline conditions × 2nd-year interactions with values of 8.48 and 8.04 mg·g^−1^ FW, respectively). Also, results related to the effects of cultivar × year interaction (Fig. 6b) explained that the maximum value for T Chl (equal to 10.86 mg·g^−1^ FW) was recorded for Chamran-2 cultivar × measuring 1st-year, while there was no significant difference with T Chl under Mehregan × 1st-year interaction with a value of 9.81 mg·g^−1^ FW. On the other hand, the minimum significant content of T Chl (with 6.78 mg·g^−1^ FW value) was reached for the interaction of Marvdasht cultivar × 1st-year of testing.

**Fig. 7:**
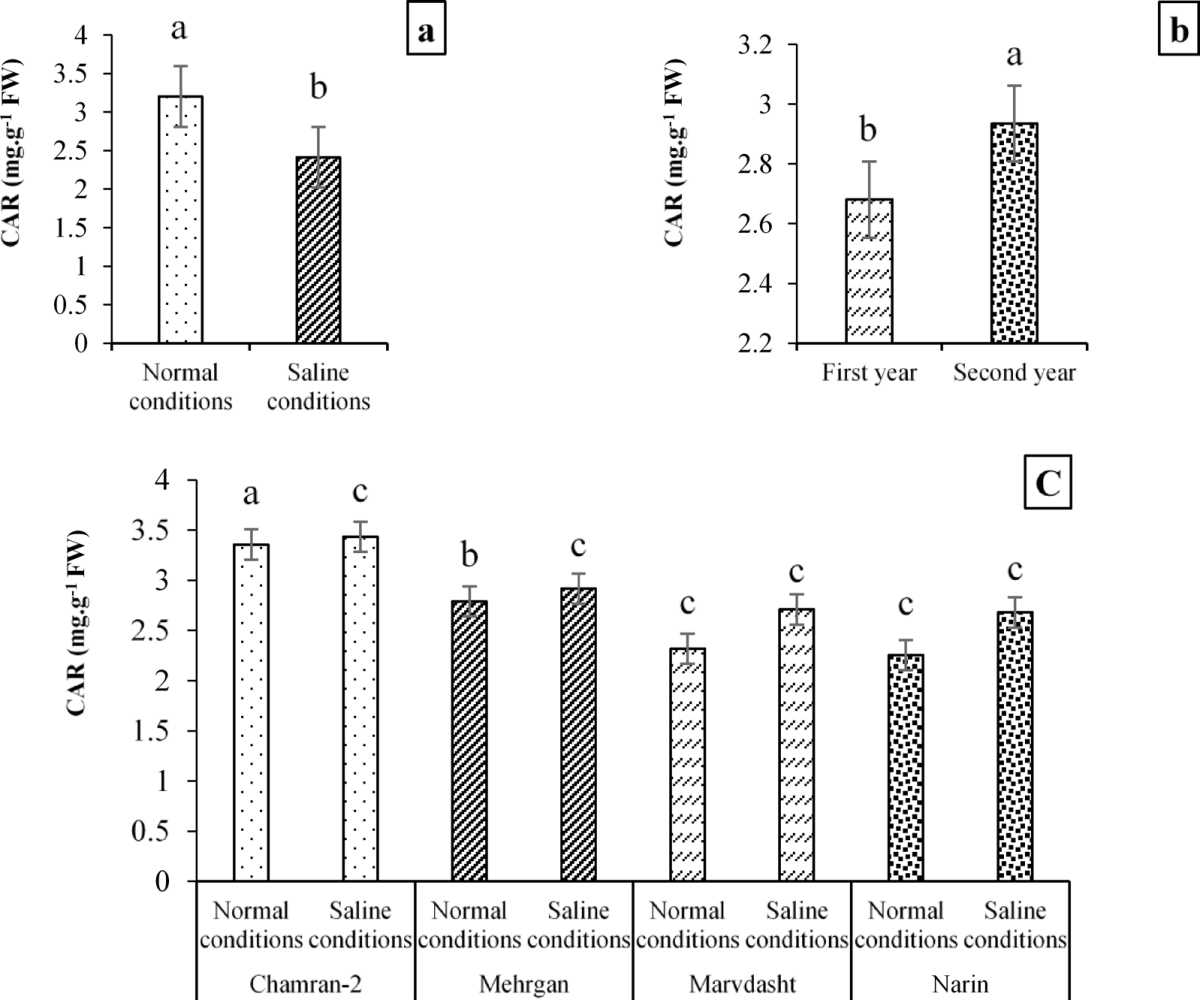
Evaluating CAR content (mean square) under field conditions (**a**), year (**b**), and cultivar × field conditions interaction (**c**). Means sharing the same superscript are not significantly different from each other at *p* < 0.05.

Figure 7 data (7a and 7b) showed that Normal field (with 3.20 mg·g^−1^ FW) compared to saline conditions (with 2.41 mg·g^−1^ FW) and the first year data (2.68 mg·g^−1^ FW) than the second year of the experiment (2.94 mg·g^−1^ FW) had significant increases for the content of CAR equal to 32.78 and 9.7%, respectively. In addition, the interaction between different cultivars and field conditions indicated notable differences among them, so that the highest content of CAR (4.17 mg·g^−1^ FW) was for the interaction of the Chamran-2 cultivar × Normal farm, which had significant differences with other interactions. Contrastingly, the results indicated minimum content of CAR was recognized for Chamran-2 cultivar × saline conditions interaction with an average value of 2.28 mg·g^−1^ FW (Fig. 7c).

#### Enzymatic (CAT and SOD) and non-enzymatic (LPC and SS) activities

The CAT activity was significantly affected by field conditions at *p* < 0.01 and year at *p* < 0.05, while the enzymatic activity of SOD was influenced by field conditions, year, cultivar, and the interaction of cultivar × field conditions × year at *p* < 0.01. Plus, despite the significant effects of field conditions and cultivar treatments on LPC at *p* < 0.01 and *p* < 0.05, respectively, year treatment and all interactions between the studied treatments had no notable effects on this index. Furthermore, the main treatment effects of field conditions, year, and cultivar at *p* < 0.01 and the interaction between field conditions and year at *p* < 0.05 were significant on the SS trait (Table 9).

**Fig. 8:**
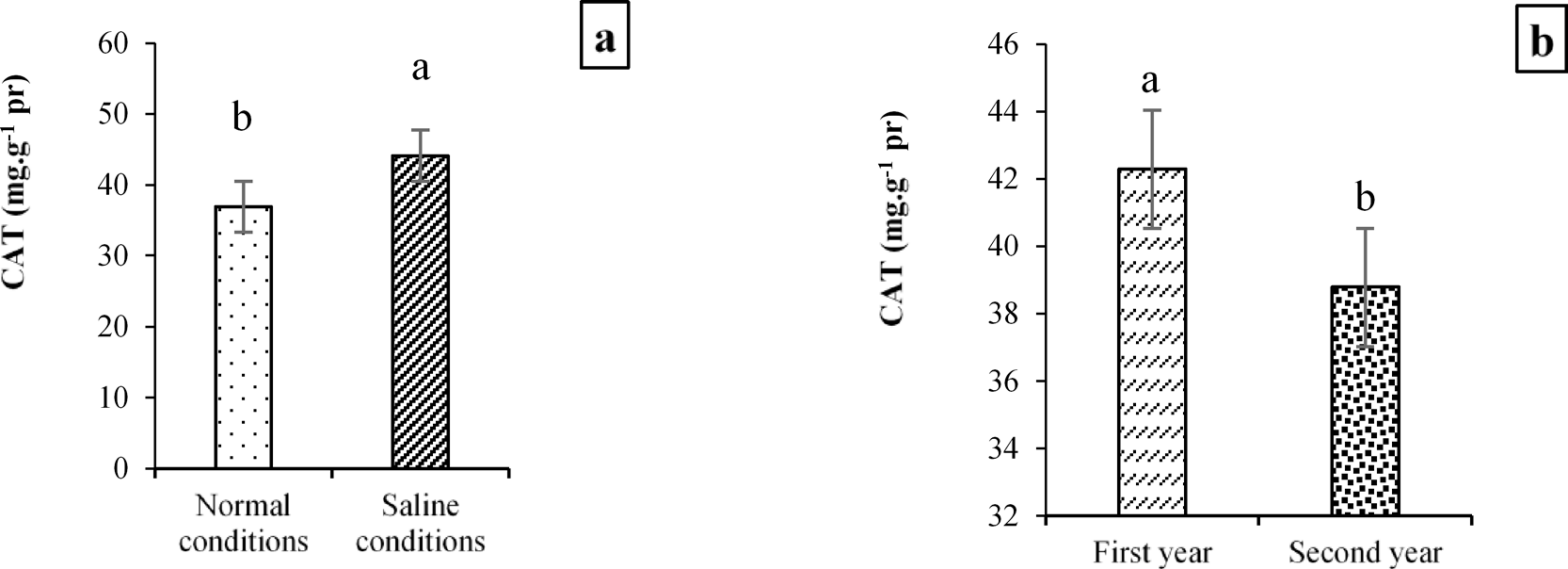
Evaluating CAT activity (mean square) under field conditions (**a**) and year (**b**) treatments. Means sharing the same superscript are not significantly different from each other at *p* < 0.05.

**Fig. 9:**
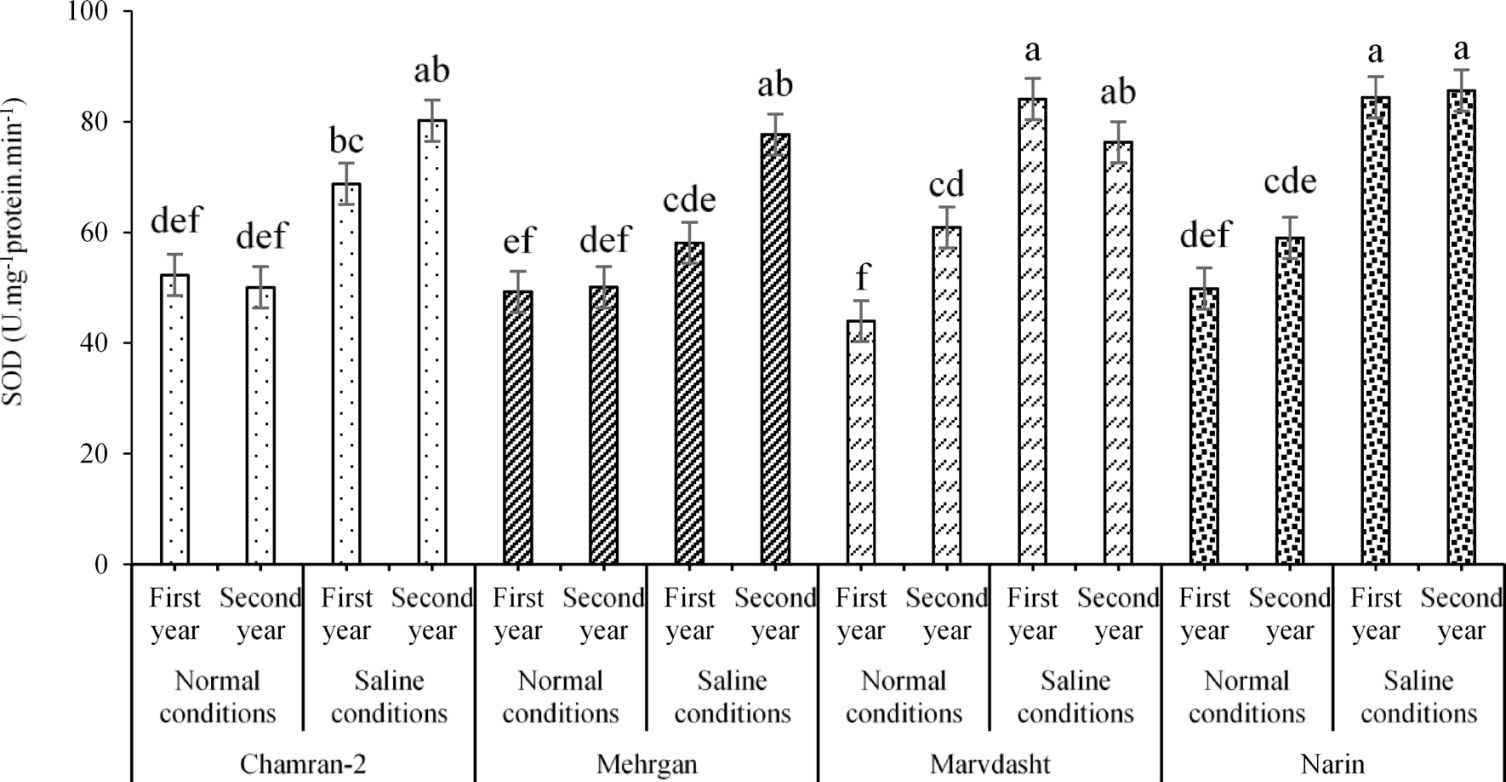
Effects of the interaction of cultivar × salinity (mean square) on the enzymatic activity of SOD. Means sharing the same superscript are not significantly different from each other at *p* < 0.05.

Results related to the effects of field conditions and year on CAT activity are shown in Fig. 6a and b. According to the data (Fig. 8a), the CAT activity in plants exposed to salinity stress (44.13 mg·g^−1^ pr) significantly increased by 16.76% compared to plants grown under normal field conditions (36.94 mg·g^−1^ pr). Furthermore, CAT activity in plants grown during the first year of the experiment (42.29 mg·g^−1^ pr) was significantly higher by 9.05% than in plants grown during the second year (38.78 mg·g^−1^ pr; Fig. 8b). In addition, since enzymatic activity of SOD was significantly affected by the interaction of cultivar × salinity, the highest and lowest activities were observed in the Narin cultivar under saline conditions (85.62 U·mg^−1^ protein·min^−1^) and the Marvdasht cultivar under normal conditions (43.97 U·mg^−1^ proteinmin^−1^), respectively (Fig. 9).

**Fig. 10:**
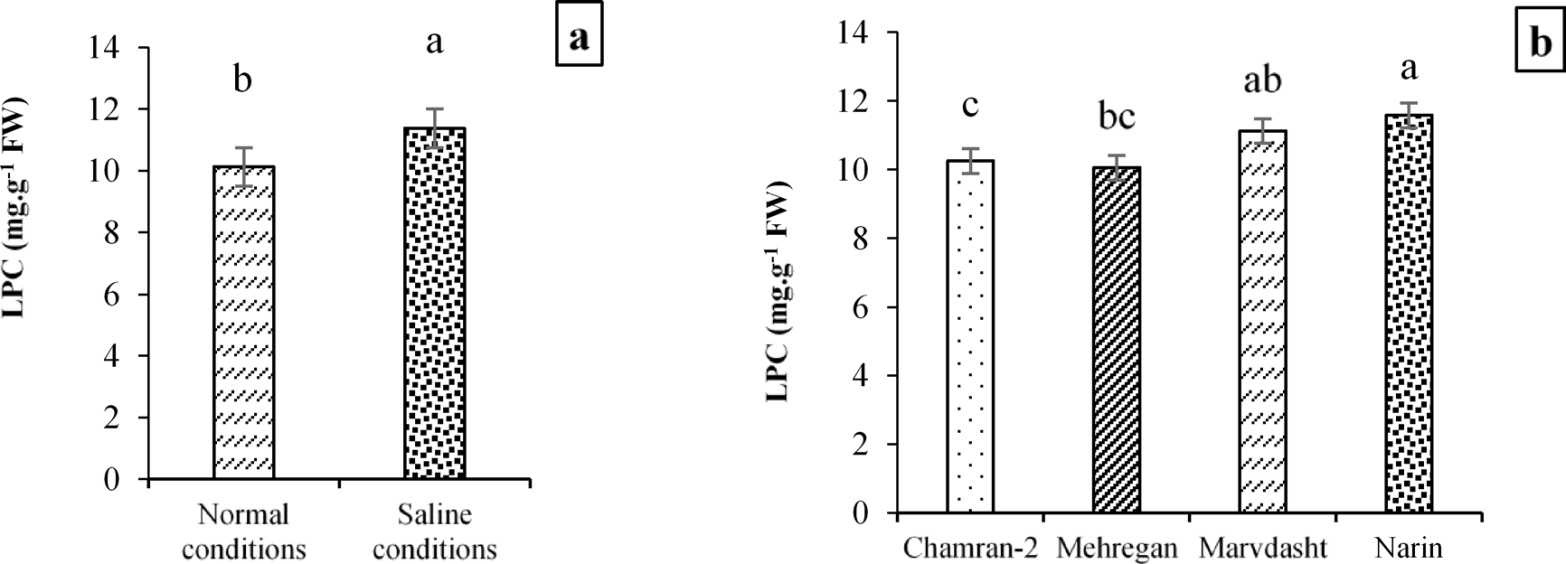
Measuring LPC trait (mean square) under field conditions (**a**) and cultivar (**b**) treatments. Means sharing the same superscript are not significantly different from each other at *p* < 0.05.

Regarding the effects of experimental treatments on non-enzymatic antioxidants, it was observed that the LPC trait was only affected by treatments of local (*p* < 0.01) and cultivar (*p* < 0.05), but there were no significant differences for year treatment and interactions studied in the present study (Table 9). Mean comparison of results confirmed a significant increase equal to 8.25% for LPC trait under the application of saline conditions (with 11.38 mg·g^−1^ FW) instead of Normal field with 10.13 mg·g^−1^ FW (Fig. 10). Moreover, the Narin cultivar (with 11.58 mg·g^−1^ FW) had notable enhances equivalent to 12.98, 15.22, 4.14% compared to Chamran-2, Mehregan, and Marvdasht cultivars for the LPC trait. On the contrary, the minimum LPC was obtained for the Mehregan cultivar (10.05 mg·g^−1^ FW), which had no notable increase over LPC in the Chamran-2 cultivar (10.25 mg·g^−1^ FW) (Fig. 10).

**Fig. 11:**
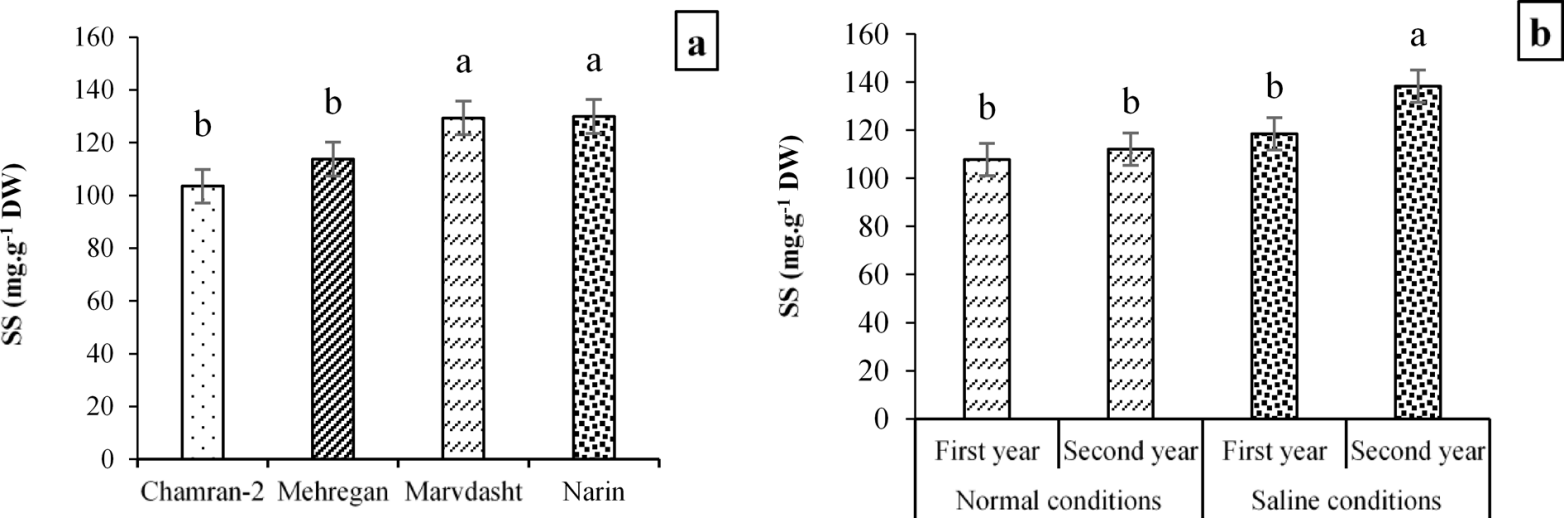
Measuring SS content (mean square) under the application of cultivar treatment (**a**) and field conditions × year interaction (**b**). Means sharing the same superscript are not significantly different from each other at *p* < 0.05.

Among studied cultivars, Narin cultivar had the highest amount in soluble sugars, which compared to Chamran-2, Mehregan, and Marvdasht cultivars had significant increases of 25.54, 14.18, and 0.42%, respectively (Fig. 11). Also, investigating effects of field conditions × year interaction on SS trait confirmed that the highest and lowest values was obtained for the interactions of saline conditions × 2nd-year (138.3 mg·g^−1^ DW) and Normal field × 1st-year (107.76 mg·g^−1^ DW). Expressly, it appears that the content of SS for the interaction of saline conditions × 2nd-year had increased compared to Normal field × 1st year, Normal field × 2nd year, and saline conditions × 1st year by 28.34, 23.35, and 16.71%, respectively (Fig. 11).

#### Qualitative indices, including grain protein (GPr), wet gluten (WG), and gluten index (GI)

The combined analysis of variance (Table 5) revealed that both GPr and GI were significantly affected by field conditions and cultivar (at *p* < 0.01 and *p* < 0.05, respectively), while the year factor and all interaction effects had no significant impact on either index. Additionally, the WG index was significantly influenced by field conditions, year, and cultivar (*p* < 0.01), as shown in Table 9.

The mean comparison of the combined data (Table 8) indicated that GPr under normal field conditions increased significantly by 12.37% compared to GPr under saline conditions. Furthermore, although the difference in GPr values between the first and second year was not statistically significant, the GPr content was higher in the first year. Among the studied cultivars, the highest and lowest GPr values (11.51 and 10.35 mg·g^−1^ FW) were observed in Chamran-2 and Narin, respectively (Table 8).

Regarding WG, plants grown under normal field conditions showed a statistically significant increase of 24.06% compared to those grown under saline conditions. Additionally, WG values in the second year of the experiment were 9.71% higher than in the first year of the study. Among the cultivars, Mehregan and Marvdasht recorded the highest and lowest WG values (33.46% and 27.68%), respectively. Overall, as shown in Table 8, there were no significant differences in WG percentage between Mehregan and Chamran-2 (the cultivars with the highest gluten content), or between Marvdasht and Narin (the cultivars with the lowest gluten content). Finally, since GI was significantly affected by both field conditions and cultivar, it was observed that plants grown under normal conditions exhibited a 16.37% increase in GI compared to those grown under saline conditions. Furthermore, the GI of the Mehregan cultivar was 8.00%, 21.74%, and 7.04% higher than that of Chamran-2, Marvdasht, and Narin cultivars, respectively (Table 8).

#### Correlation coefficients

The Pearson’s correlation coefficients among the measured traits in the first year are presented in Table 9. Total chlorophyll content (Chl T) showed a strong and significant positive correlation with grain yield (GY) (r = 0.7516, *p* = 0.0048*) and thousand grain weight (TGW) (r = 0.6433, *p* = 0.0240*). Similarly, carotenoid content (CAR) exhibited a highly significant positive correlation with biological yield (BY) (r = 0.8447, *p* = 0.0005*) and TGW (r = 0.7378, *p* = 0.0062*). In contrast, antioxidant enzyme activities were negatively correlated with yield-related parameters. Superoxide dismutase (SOD) activity was strongly and negatively associated with BY (r = − 0.8076, *p* = 0.0015*), while catalase (CAT) activity also showed a negative correlation with BY (r = − 0.6156, *p* = 0.0331*). A significant positive correlation was observed between BY and GY (r = 0.6850, *p* = 0.0492*), indicating a close relationship between total biomass and final grain yield (Table 10).

**Table 10:** Pearson correlation coefficients among early-stage physiological, biochemical, and yield-related traits of wheat under salinity conditions (according to the first-year data).

SOV	Chl T	CAR	SS	LPC	CAT	SOD	TGW	BY	GY
Chl T	1.00								
CAR	0.5860 0.0452	1.00							
SS	− 0.4304 0.1630	− 0.4902 0.1057	1.00						
LPC	− 0.3705 0.2358	− 0.4120 0.1831	− 0.1076 0.7392	1.00					
CAT	− 0.4608 0.1316	− 0.7156 0.0089	0.1869 0.5607	0.4850 0.1100	1.00				
SOD	− 0.6047 0.0372	− 0.4361 0.1564	0.0488 0.8803	0.4855 0.1095	0.4873 0.1081	1.00			
TGW	0.6433 0.0240	0.7378 0.0062	− 0.5024 0.0960	− 0.5093 0.0908	− 0.3551 0.2574	− 0.2809 0.3764	1.00		
BY	0.4779 0.1160	0.8447 0.0005	− 0.1675 0.6029	− 0.6156 0.0331	− 0.8076 0.0015	− 0.6056 0.0369	0.4730 0.1204	1.00	
GY	0.7516 0.0048	0.4928 0.1036	− 0.2959 0.3503	0.0319 0.9215	− 0.2839 0.3711	− 0.3273 0.2990	0.3635 0.2454	0.6850 0.0492	1.00

Also, the Pearson’s correlation coefficients in the second experimental year (Table 11) indicated that Chl T showed a strong and significant positive correlation with biological yield (BY) (r = 0.8892, *p* = 0.0001*) and a significant positive relationship with GY (r = 0.6804, *p* = 0.0149*). The CAR content was also highly and positively correlated with GY (r = 0.9138, *p* < 0.0001*) and BY (r = 0.7316, *p* = 0.0068*). A strong negative correlation was found between soluble sugars (SS) and Chl T (r = − 0.9002, *p* < 0.0001*), CAR (r = − 0.6802, *p* = 0.0149*), and yield traits (BY: r = − 0.7855, *p* = 0.0025*; GY: r = − 0.7494, *p* = 0.0050*). Likewise, antioxidant enzyme activities (CAT and SOD) were significantly and negatively correlated with yield-related parameters, including BY (CAT: r = − 0.7884, *p* = 0.0023*; SOD: r = − 0.6386, *p* = 0.0245*). Positive relationships were again observed between yield components, where BY was significantly correlated with GY (r = 0.7240, *p* = 0.0078*).

**Table 11:** Pearson correlation coefficients among early-stage physiological, biochemical, and yield-related traits of wheat under salinity conditions (according to the second-year data).

SOV	Chl T	CAR	SS	LPC	CAT	SOD	TGW	BY	GY
Chl T	1.00								
CAR	0.6658 0.0181	1.00							
SS	− 0.9002 < .0001	− 0.6802 0.0149	1.00						
LPC	− 0.2756 0.3860	− 0.3196 0.3112	− 0.3924 0.2071	1.00					
CAT	− 0.6923 0.0126	− 0.5455 0.0666	0.6337 0.0269	0.3666 0.2412	1.00				
SOD	− 0.7493 0.0050	− 0.5887 0.0440	0.6958 0.0120	0.2667 0.4020	0.7342 0.0065	1.00			
TGW	0.5384 0.0709	0.4999 0.0979	− 0.6418 0.0245	− 0.6162 0.0329	− 0.4102 0.1854	− 0.6350 0.0265	1.00		
BY	0.8892 0.0001	0.7316 0.0068	− 0.7855 0.0025	− 0.3260 0.3011	− 0.7884 0.0023	− 0.6386 0.0245	0.3140 0.3203	1.00	
GY	0.6804 0.0149	0.9138 < .0001	− 0.7494 0.0050	0.1603 0.6186	− 0.4723 0.1211	− 0.5283 0.0774	0.4313 0.1616	0.7240 0.0078	1.00

## Discussion

Salinity in arid and semi-arid regions is a leading threat and one of the most important direct or indirect factors affecting seed germination and different stages of growth, development, and productivity of plants in soil and other environments, as was previously confirmed by several researchers^15,69–74^. On the other hand, seed germination is known as the most vital and susceptible phase in seedling establishment that can determine successful crop production and future plant growth and development^75–77^. Several studies have also reported the negative effects of salinity on seed germination. These effects vary among plant species and wheat genotypes, as well as plant growth and development, grain yield, and other quantitative and qualitative characteristics through reducing water availability, increasing nutrient imbalance, ROS-mediated oxidative stress, and enhancing ion toxicity (a long-term effect that associated with over-production of singlet oxygen, superoxide ion, H_2_O_2_, and other radicals, as well as absorbing excessive Cl^−^ and Na^+^); however, the above adverse effects varied depending on the plant species/genotype, environment, and their interaction^72,75^. Our results from Petri dish experiments revealed significant variability among wheat cultivars and promising lines in germination indices (GP, T50, MTG, SG) under salinity. Chamran-2 exhibited the highest GP and lowest T50, indicating a superior capacity to overcome osmotic stress and initiate germination, which reflects its inherent tolerance mechanisms. Enhanced germination in tolerant genotypes may result from more efficient osmotic adjustment, ion compartmentalization, and early activation of antioxidant systems. Roots, being the first organs exposed to salinity, play a critical role in stress sensing and resource acquisition. In addition, since roots are the first organs to be exposed to different levels of salinity stress, a reduction in root length and plant height is a common phenomenon in many plants under salt stress conditions; therefore, the root length is known as a critical index for assessing stress tolerance and directly affects nutrient and water uptake, and other soil-linked stresses are first sensed by seeds and plant roots^31,75,78–83^. Researchers also declared that salinity stress first affects the root system through osmotic stress and reduced water uptake, resulting in ionic toxicity in plants due to nutrient imbalance, Na toxicity, and growth inhibition^84,85^. Hence, the Root Length Stress Tolerance Index (RLSI) can provide a standardized measure of root growth for saline environments^86,87^. On this subject, some other studies announced that assessing indices relevant to plant roots could be considered to improve resource capture and plant development under adverse and stressful situations^88–90^. As can be seen (Tables 3 and 4), variations among all salinity levels were significant for indices of SL, RL, RLSI, and LPr in the Petri test, where increasing salinity significantly reduced their values for six bread wheat cultivars/new promising lines. In our study, tolerant genotypes maintained longer roots and higher RLSI, supporting their ability to explore soil efficiently and access water and nutrients under saline conditions^91,92^.

According to our findings, cultivars of Chamran-2 and Mehregan illustrated greater tolerance to salinity and better growth and development qualifications (Table 8). Shafi et al.^93^ reported that salt tolerance varies among different wheat genotypes, and the degree of tolerance to salinity is attributed to the greater capacity for acclimation in superior wheat genotypes. In this regard, it was also explained that salinity affects plants via stressful impacts of osmotic stress, ionic toxicity, metabolic changes, reduction of cell division, and subsequently, influences plants’ growth and development stages^94^. Accordingly, decreasing indicators related to germination and other growth indicators in the present study can be attributed to the adverse effects of salinity stress on the root system and RLSI, which were confirmed by previous studies. In this case, reducing indices of growth rate, root length, root/shoot ratio, and smaller and fewer leaves were also reported by Imran et al.^95^ as common influences of salinity stress. It has also been reported that plant growth and development responses to the adverse effects of salt stress might be dependent on lower Na⁺ and Cl^–^ and higher K^+^ uptake in genotypes with more salt-tolerant abilities^96,97^. Therefore, monitoring genetic diversity among genotypes is important for understanding the mechanisms of salt tolerance and for improving salt tolerance. Our findings on germination-related indices confirmed that Chamran-2 and Mehregan genotypes can be considered salt-tolerant and should be given more attention in future experiments. Although preliminary Petri dish experiments included both varieties and promising lines, only the four superior varieties were selected for the field trials under Iranian conditions, as the lines did not show satisfactory performance in preliminary tests. This decision allowed us to focus the field evaluation on genotypes with higher potential under the tested conditions.

Field experiments further demonstrated that environmental conditions (salinity and year) and genotype influenced morphological, biochemical, and physiological traits (Tables 7, 8 and 9). In general, the activity of antioxidant enzymes such as CAT and SOD increased under saline conditions, reflecting an adaptive response to ROS accumulation. Strong positive correlations among CAT, SOD, and yield traits suggest that early-stage antioxidant capacity is crucial for protecting cellular structures and sustaining productivity under stress. Conversely, traits such as soluble sugar accumulation correlated negatively with yield, indicating that excessive sugar may signal stress severity rather than tolerance. These findings highlight the importance of integrating biochemical markers with morphological traits to assess salinity tolerance comprehensively. Some researchers have demonstrated that salinity affects the percentage of seedling emergence, the length of the root and shoot, the dry weight of the root and shoot, the relative water content, and the enzymatic activities of catalase, peroxidase, and superoxide dismutase^98^. Also, some other researchers have shown that salinity environments can reduce plant height, plant biomass, grain protein, net photosynthetic rate, transpiration rate, stomatal conductance, photosynthetic pigments, and grain yield while increasing enzymatic and non-enzymatic activities of CAT, SOD, POX, carbohydrates, and proline in plants^20,96,99,100^. It was also reported that reducing salinity-induced photosynthetic pigments can occur by decomposition of chlorophyll enzymes^101^, enzyme activities (e.g., glutathione reductase), and reducing oxidative stress-induced ALA (5-Aminolevulinic acid) synthesis, which plays a crucial role in associating abiotic/environmental stress tolerance in different plants^102–106^. Previously, it was found that chlorophyll conservation, carotenoids, and their stability are the main parameters associated with salinity tolerance in plants and are inversely related to salinity increase^20,107^. Also, we found that photosynthetic pigments and carotenoids for different genotypes decreased in saline conditions (Table 8; Figs. 3, 4, and 5). Therefore, we concluded that reducing chlorophylls and carotenoids among different studied cultivars can be due to unfavorable growth conditions and oxidative or secondary stresses caused by high salinity in the salt conditions. These outcomes are supported by some researchers^108,109^.

Table 8 confirmed that improving traits of GPr, WG, and GI in Chamran-2 and Mehregan cultivars were more than those of Marvdasht and Narin. Also, the mentioned traits in the normal field had a significant increase compared to the saline conditions. It was also confirmed that the adverse effects of salinity levels on morphophysiological, grain quality (such as wet gluten and gluten index), grain yield, and yield-related components of wheat cultivars were varied and depended on cultivars’ salinity tolerance and their different genetic potential^109–112^. Regarding quality indices, the results demonstrated that both genotype and stress treatments (salinity and drought) had significant effects on all grain quality-related traits. Notably, the impact of salinity stress was more pronounced than that of drought stress^113^. In line with the current study, stress conditions significantly reduced thousand-grain weight and grain protein yield. However, unlike our findings, previous research reported a significant increase in wet gluten content under stress conditions. Nevertheless, Zheng et al.^114^ observed that wet-gluten concentration, gluten index, and grain protein decreased in two cultivars of winter wheat and were consistent with the present study issues. In another study, it was proved that salinity stress decreased relative water content, chlorophyll contents, CAR, biomass, and grain yield, but increased proline, soluble sugars, superoxide dismutase, and catalase activity^72,115–117^. Plus, salinity impacts and values of morphological, biochemical, and functional traits in the two cultivars were significantly different from each other. In general, the offered results by these researchers were in line with our findings. In addition, due to the ionic similarity and the high tendency of Na^+^ to replace K^+^, many enzyme activities linked to K^+^ are disrupted, K^+^ concentration and K^+^:Na^+^ ratio are decreased, and some critical plant processes, e.g., photosynthesis and chlorophyll synthesis, are influenced by the Na^+^-induced toxicity^96,118,119^.

Our findings also indicated that GY and its components were significantly reduced under salinity conditions, but Chamran-2 and Mehregan outperformed Marvdasht and Narin in terms of plant height, thousand-grain weight, biomass, and grain yield. Several researchers have also noted that GY is commonly used as a primary indicator of salt tolerance in wheat^121,122^. Therefore, according to the latter researchers’ statements, it can be concluded that cultivars with higher yields (here, Chamran-2 and Mehregan cultivars) were more tolerant to salinity stress. In this regard, the laboratory results (Tables 3 and 4) also determined that Chamran-2 and Mehregan cultivars had the best germination conditions and germination-related indices. Supporting the above findings, it has been reported that plants tend to produce lower yields under salinity stress due to its detrimental effects on relative water content, total dry weight, plant height, and leaf number, mainly through reduced water potential, ion toxicity, and ionic imbalance^123^. Similarly, Mousavi et al*.*^124^ reported a positive and direct correlation between grain yield and traits such as plant height, thousand-seed weight, and biomass. In agreement with these findings, the results of the present correlation analysis across both experimental years (Tables 10 and 11) revealed that several early-stage physiological and biochemical traits were strongly associated with yield-related parameters. Notably, Chl T and CAR contents measured during the vegetative stage showed significant positive correlations with BY and GY (r ≈ 0.87–0.91, *p* < 0.01), suggesting that genotypes maintaining higher pigment concentrations under saline conditions can preserve photosynthetic efficiency and sustain higher productivity. Conversely, the SS trait displayed strong negative correlations with both BY and GY (r ≈ − 0.74 to − 0.79, *p* < 0.01), indicating that sugar accumulation reflects stress severity rather than tolerance potential. Antioxidant enzymes, particularly CAT and SOD, were positively correlated with each other (r ≈ 0.73, *p* < 0.01) and moderately linked to yield traits, highlighting their complementary roles in mitigating oxidative damage under salt stress.

These patterns provide strong empirical support for the assumption that parameters measured in early growth stages, especially photosynthetic pigments and antioxidant enzyme activity, are significantly correlated with performance at later developmental stages and with final yield. Among the early-stage traits, pigment stability (i.e., chlorophyll and carotenoid retention) appears to be a robust and practical indicator of salinity tolerance, as it mirrors the plant’s ability to maintain ionic and oxidative balance and thus contributes to improved biomass and grain yield. This observation aligns with Omrani et al.^125^, who reported high heritability for chlorophyll and carotenoid traits under salinity stress, and Pastuszak et al.^126^, who confirmed that these pigments effectively distinguished salt-sensitive and salt-tolerant wheat accessions. Therefore, early-stage measurements of chlorophyll and carotenoids may serve as reliable, non-destructive physiological markers for the rapid identification of salt-tolerant wheat genotypes, thereby enhancing breeding efficiency and reducing the duration of field evaluation.

## Conclusions

Identifying and selecting the most salt-tolerant wheat varieties is essential for success in agricultural activities. In this study, we found significant differences among wheat genotypes under saline conditions during germination, plant growth, and development stages, as well as in the field-tested traits, including morphophysiological, biochemical, and yield-related characteristics, to identify superior genotypes. The results showed that while salinity negatively affected all traits, cultivar treatment had no significant impact on GP and MTG indices. Conversely, in field experiments, both field conditions and cultivar significantly influenced all indices (except CAT activity under cultivar treatment). Additionally, there were no significant differences in BY, PC, GPr, and GI traits between the two experimental years. Our findings indicated that Chamran-2 and Mehregan genotypes showed acceptable salt tolerance compared with the other studied genotypes, indicating that they can be valuable and suitable resources for cultivation in saline-affected regions. Also, among the early growth stage traits, germination percentage (GP), mean time to germination (MTG), and leaf proline content (LPC) were identified as the most adequate parameters for evaluating salinity tolerance in wheat, providing useful indicators for early selection of tolerant genotypes. Furthermore, correlation analyses indicated that early-stage physiological traits, particularly chlorophyll and carotenoid contents, were positively associated with later-stage yield parameters, reinforcing their value as reliable markers for selecting salt-tolerant wheat genotypes. Monitoring these traits can thus support rapid and efficient early-stage screening, complementing field evaluations. Overall, given that these genotypes exhibited more favorable plants, heavier grain weight, and higher grain and biological yields, we recommend that farmers in the study area and similar regions adopt and cultivate the Chamran-2 and Mehregan cultivars.

## Data Availability

The datasets generated, used, deployed, and analyzed during the present study are not accessible to the general public. However, they are available from the corresponding author upon adequate request.

## Abbreviations

BY: Biological yield
CAR: Carotenoids
CAT: Catalase
Chl a: Chlorophyll a
Chl b: Chlorophyll b
Chl T: Total chlorophyll
CV: Coefficient of variation
DW: Dry weight
EDTA: Ethylenediamine tetraacetic acid
FW: Fresh weight
GI: Gluten index
GP: Germination percentage
GPr: Grain protein
GY: Grain yield
LPC: Leaf proline content
LPr: Leaf protein
MTG: Mean time to germination
PH: Plant height
RL: Root length
RLSI: Root length stress tolerance index
SG: Speed of germination
SL: Shoot length
SOD: Superoxide dismutase
SOV: Source of variation
SS: Soluble sugars
SY: Straw yield
T_50_: Time to reach 50% of final/maximum germination
TGW: 1000-Grain weight
WG: Wet gluten content
